# A built-in self-calibrating luminescence sensor based on RhB@Zr-MOF for detection of cations, nitro explosives and pesticides†

**DOI:** 10.1039/d0ra02843f

**Published:** 2020-05-20

**Authors:** Liu Yang, Yu-Long Liu, Cheng-Guo Liu, Ying Fu, Fei Ye

**Affiliations:** Department of Applied Chemistry, College of Science, Northeast Agricultural University Harbin 150030 People's Republic of China fuying@neau.edu.cn yefei@neau.edu.cn +86-451-55190930; Department of State Assets Management, Northeast Agricultural University Harbin 150030 People's Republic of China

## Abstract

A RhB@Zr-MOF composite with dual-emission properties was successfully constructed, which comprises a zirconium-based metal–organic framework and the luminescent dye molecule, Rhodamine B (RhB), embedded *via* the encapsulation method. The fluorescence intensity ratio of the two emissions was found to be *ca.* 370 nm/590 nm for RhB@Zr-MOF. The fluorescence intensity values of the two emissions of RhB@Zr-MOF can also be affected by the structures of analytes containing different organic groups. Due to the existence of the dual-emission properties in RhB@Zr-MOF, the relative fluorescence intensity of the emission peaks was introduced as a detection index instead of absolute fluorescence intensity. RhB@Zr-MOF, which possesses the characteristics of a built-in self-calibrating fluorescence sensor, was investigated for detecting cations, nitroaromatics and pesticides. Aside from high sensitivity and selectivity, recyclability is the most important property for sensing pesticides. This work shows that RhB@Zr-MOF can maintain its stability after 5 cycles of detecting nitenpyram, with LOD of 0.2 μM. These results demonstrate that dye@MOFs with dual-emission properties can be employed as multifunctional fluorescence sensors for different types of analytes, and that RhB@Zr-MOF provides a new paradigm for analyte sensing.

## Abstract 2

A RhB@Zr-MOF composite with dual-emission properties was demonstrated as a self-calibrating sensor for cations, nitro explosives and nitenpyram.

## Graphical abstract

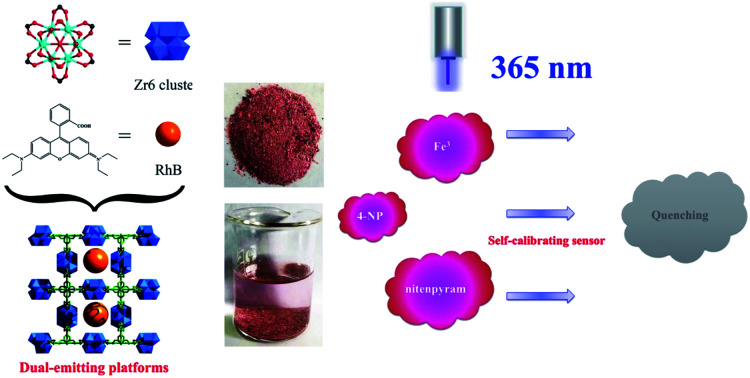

## Introduction

Pesticides,^1–3^ a general term for toxic organic compounds used in agriculture, play a crucial role in agricultural production worldwide. However, pesticide residues can threaten human health, food safety and ecosystems.^4–6^ On the basis of the problems mentioned above, technologies that can detect pesticides effectively and sensitively are particularly desirable compared with classical methods, such as gas chromatography (GC),^7^ high-performance liquid chromatography (HPLC)^8^ and mass spectrometry (MS),^9^ which are costly, involve tedious sample preparation procedures and complex instrumental methods, and require highly skilled labor power.^10–12^

Explosives or explosives-like toxic molecules, which exist everywhere in our daily life, are associated with issues of human health, national defense, industrial production and environmental protection.^13,14^ When storing or transporting explosives, flames or high temperatures are absolutely not allowed. Meanwhile, it is necessary to avoid friction between explosives. Because of the hazards associated with explosives and explosives-like toxic molecules, developing sensitive, accurate and cost-effective methods of detecting them is essential. Many conventional materials have been selected to detect them, such as nanoscale materials and some polymers.^15,16^ So far, fluorescence sensors have become the most effective technology for detection, as they possess the merits of sensitivity and rapidness.

Detecting trivalent metal ions (Fe^3+^, Cr^3+^, Al^3+^ and Ga^3+^) is extremely vital because of their deposition in various biological systems.^17,18^ In comparison with conventional techniques for sensing trivalent metal ions, such as chromatography, accelerator mass spectroscopy (AMS), flame atomic absorption spectroscopy (FAAS), and inductively coupled plasma mass spectrometry (ICP-MS), chemical sensors, especially fluorescence sensors, have been proposed as a promising candidate.^19–21^ Fluorescence sensors for detecting trivalent metal ions have the merits of simplicity, rapidness, sensitivity and low limits of detection (LODs), allowing them to overcome the drawbacks of the conventional methods.

Metal–organic frameworks (MOFs), also known as porous coordination polymers, are hybrid molecular networks comprising functional organic ligands and metal ions or metal clusters formed *via* a self-assembly procedure.^22–24^ MOFs appear in many fields, such as chemosensing,^25,26^ gas adsorption and storage,^27,28^ catalysis^29,30^ and so on. However, most porous MOFs are sensitive to moisture, which limit their applications in diverse fields.^31^ Hence, fabricating MOFs with high chemical and thermal stabilities is a great challenge. Recently, Zr-based MOFs have been considered as high stability materials, where zirconium ions are in the +4 valence state and exhibit high charge density and bond polarization. When zirconium ions are combined with oxygen atoms of carboxylate, they exhibit strong electron affinity, which makes Zr-MOFs extremely stable in aqueous solution and organic solvents.^32^ Fluorescence sensors based on functional MOFs synthesized *via* post-synthetic modification have been widely utilized in sensing small molecules.^33–35^ Such MOFs feature a single emission, with the drawback of low recognition and stability. In order to solve these two problems, luminescent dye molecules and Zr-MOFs have been integrated together to construct dye@Zr-MOFs that display dual-emission properties.^36^ The integration broadens the spectral range and increases the types of analytes that can be detected. The overlap of the spectra of Zr-MOFs and luminescent dye molecules creates a novel type of fluorescence sensor with energy transfer in the direction of MOFs to dye molecules.^37^ In the production of self-calibrating fluorescence sensors, the relative fluorescence intensity of emission peaks can be used as a detection index instead of absolute fluorescence intensity.^38^ Dual-emission materials have successfully exhibited improved detection accuracy while avoiding unstable instrumental parameters, which can affect the final luminescence intensity. In recent years, many luminescence sensing systems based on MOFs and dye molecules have been reported.^39–42^ The number of dye@MOF sensors with dual-emission properties is growing constantly.

In this work, we have selected 1,3,5-benzenetribenzoic acid (BTB), and ZrCl_4_ to prepare a luminescent Zr-MOF (CCDC: 1000802)^43^ containing nodes of Zr6 clusters. Furthermore, RhB@Zr-MOF was fabricated by encapsulating the luminescent dye molecule, Rhodamine B (RhB), into Zr-MOFs. RhB@Zr-MOF shows a dual-emission luminescence property, and acts as a multifunctional, self-calibrating fluorescence sensor for detecting cations, nitro explosives and pesticides. Due to the energy-transfer interactions between the host Zr-MOF and the guest RhB, the RhB@Zr-MOF composite can selectively detect Fe^3+^, 4-NP and nitenpyram by measuring the relative fluorescence intensities of two emissions instead of their absolute fluorescence intensities. The fluorescence intensity changes mainly come from Zr-MOF, with the calibration of the relatively weak intensity of encapsulated RhB molecules. A range of methods, including thermogravimetric analyses (TGA), powder X-ray diffraction (PXRD), and infrared radiation (IR), have been employed to characterize RhB@Zr-MOF. RhB@Zr-MOF displays high thermal stability, recyclability and low LODs for sensing various analytes, especially nitenpyram. The mechanism for detecting nitenpyram was also investigated thoroughly towards expanding the scope of RhB@Zr-MOF.

## Experimental

### Materials and physical measurements

Zirconium(iv) chloride (ZrCl_4_, 99.5%) and 1,3,5-benzenetribenzoic acid (BTB) were purchased from Alfa. Rhodamine B (AR) was purchased from Sigma-Aldrich. KCl, NaCl, AgNO_3_, CuCl, PbCl_2_, MgCl_2_, CoCl_2_, CdCl_2_, BaCl_2_, HgCl_2_, MnCl_2_, CuCl_2_, CaCl_2_, NiCl_2_, FeCl_2_, AlCl_3_, FeCl_3_, CrCl_3_, GaCl_3_ and InCl_3_ were purchased from Aladdin. 1,2-dinitrobenzene (1,2-DNB), 1,3-dinitrobenzene (1,3-DNB), 1,4-dinitrobenzene (1,4-DNB), 2-nitrophenol (2-NP), 3-nitrophenol (3-NP), 4-nitrophenol (4-NP), 2-nitrotoluene (2-NT), 3-nitrotoluene (3-NT), 4-nitrotoluene (4-NT) and 2-nitro-*m*-xylene (NX) were purchased from Sigma-Aldrich. Isoxaflutole, fluroxypyr, teflubenzuron, rotenone, acetamiprid, etoxazole, carbendazim, carbaryl, thiamethoxam and nitenpyram were also purchased from Sigma-Aldrich. Ethanol, methanol, *N*,*N*′-dimethylacetamide (DMA) and *N*,*N*′-dimethylformamide (DMF) were purchased from Aladdin. All reagents in this work were commercially available and utilized without further purification.

### Characterization and instrumentation

The PXRD data were collected on a Siemens D5005 diffractometer with Cu-Kα radiation, with angles ranging from 3° to 40° at ambient temperature. IR spectra were measured on an Alpha Centaurt FT/IR spectrophotometer using KBr pellets, over a wavenumber range of 4000–400 cm^−1^. Thermogravimetric analysis (TGA) was carried out using a PerkinElmer TG-7 analyzer by heating the samples from 25 °C to 800 °C with a ramping rate of 10 °C min^−1^ under a steady flow of N_2_. N_2_ adsorption measurements were collected using a Quantachrome Autosorb-iQ. The samples were immersed in CH_2_Cl_2_ for further guest-exchange and degassed at 80 °C for 24 h for experimentation. Fluorescence spectroscopy data were gathered using an LS-55 with a xenon lamp and quartz carrier at ambient temperature. UV-vis spectra were obtained on a Shimadzu UV-2700 spectrophotometer in the wavelength range from 200 nm to 800 nm.

### Synthesis of Zr-MOF

Zr-MOF was synthesized by following the literature report with a few modifications.^43^ A mixture of BTB (88 mg, 0.20 mmol) and ZrCl_4_ (46 mg, 0.20 mmol) was placed into a mixed solvent of DMF (2 mL)/acetic acid (6 mL) and was smoothly stirred for 30 min at ambient temperature. The mixture was then poured into a 25 mL Teflon-lined autoclave, which was heated from 25 °C to 120 °C and maintained at this temperature for 72 hours. Colorless blocks of crystals were obtained after cooling the mixture to ambient temperature at a rate of 5 °C h^−1^, which were washed with H_2_O several times and dried at ambient temperature. Yield: *ca.* 80% (108 mg) yield based on Zr(iv).

### Synthesis of RhB@Zr-MOF

The mixture of BTB (88 mg, 0.20 mmol), ZrCl_4_ (46 mg, 0.20 mmol), RhB (96 mg, 0.20 mmol), DMF (2 mL) and acetic acid (6 mL) was put together and was smoothly stirred for 30 min at ambient temperature. The mixture was then poured into a 25 mL Teflon-lined autoclave, which was heated from 25 °C to 120 °C and maintained at this temperature for 96 hours. Pink powder was obtained after cooling to ambient temperature at a rate of 5 °C h^−1^, which was washed with DMF and H_2_O several times and dried at ambient temperature.

### Photoluminescence experiments

Fluorescence spectra of RhB@Zr-MOF in various solvents were studied. To prepare each sample, 3 mg of finely-ground RhB@Zr-MOF was soaked in 3 mL of DMF, DMA, methanol, ethanol or H_2_O, and each mixture was ultrasonicated to form a stable suspension.

### Sensing cations

For each sample, finely-ground RhB@Zr-MOF (3 mg) was soaked in H_2_O (3 mL) and the mixture was ultrasonicated to form a stable suspension. An aqueous solution of each of the cations, K^+^, Na^+^, Ag^+^, Cu^+^, Pb^2+^, Mg^2+^, Co^2+^, Cd^2+^, Ba^2+^, Hg^2+^, Mn^2+^, Cu^2+^, Ca^2+^, Ni^2+^, Fe^2+^, Al^3+^, Fe^3+^, Cr^3+^, Ga^3+^ and In^3+^ (20 μL), was added to each suspension sample. The final concentration of metal salts in each sample was 0.060 mM.

### Sensing nitro explosives and pesticides

The procedure of sensing nitro explosives and pesticides was almost the same as that for sensing cations, except that the aqueous solutions were replaced with ethanol solutions. The final concentrations of the samples of nitro explosives and pesticides were both 0.060 mM.

### Computational details

All calculations were performed using the Gaussian 09W software package.^44^ The geometries were optimized by the B3LYP^45,46^ functional. The 6-31G* basis set was used for all atoms. All geometry optimizations were performed without symmetry constraints in conjunction with the PCM continuum solvation model,^47^ using the most common solvent, water.

## Results and discussion

### Characterization of RhB@Zr-MOF

**Fig. 1:**
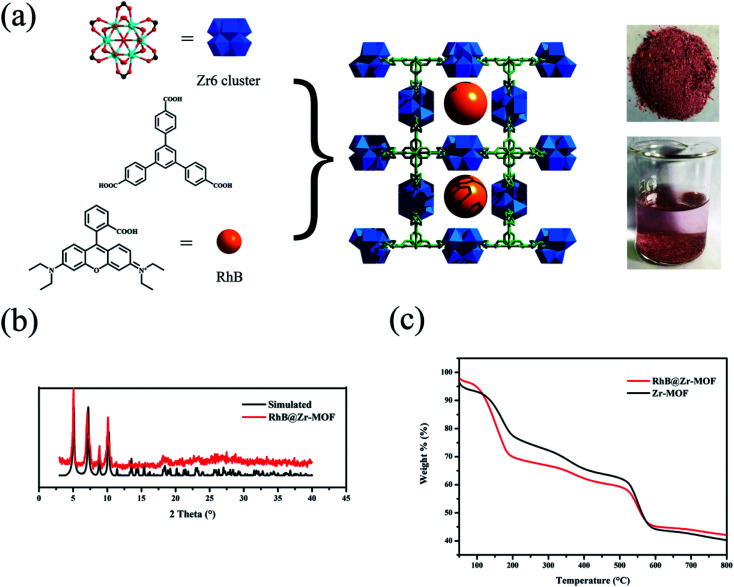
(a) The structure of RhB@Zr-MOF composite synthesized *via* a “one-pot” method. The photograph on the upper right is the solid state of RhB@Zr-MOF. The photograph on the bottom right is the solid state of RhB@Zr-MOF in H_2_O. (b) PXRD powder diffraction patterns of simulated of Zr-MOF (black) and RhB@Zr-MOF (red). (c) TG curves of Zr-MOF (black) and RhB@Zr-MOF (red).

Rhodamine B (*ca.* 15.9 Å × 11.8 Å × 5.6 Å), a cationic dye molecule with luminescence properties, has been extensively utilized in numerous fields, including printing, fiber products, cosmetics, pharmaceuticals, *etc.*^48–50^ Zr-MOFs, as robust structures, possess high chemical and thermal stabilities, making them suitable for encapsulating luminescent dye molecules. In this work, a Zr-MOF with a kdg topology, Zr_6_(μ_3_-O)_4_(μ_3_-OH)_4_(OH)_6_(H_2_O)_6_(BTB)_2_·6DMF·H_2_O, was synthesized, where the Zr6 cluster can be considered as a 6-connected node, and the BTB ligand can be simplified to a 3-connected linker. This Zr-MOF has 1D channels and the dimensions of the channels are 14.37 Å × 14.37 Å. Using a “one-pot” method, Rhodamine B molecules were loaded into the channels of Zr-MOF to form RhB@Zr-MOF, where the size of RhB was apt to fit in the channels of Zr-MOF. The color of RhB@Zr-MOF soaked in H_2_O was pink compared with the white color of Zr-MOF, which demonstrated that RhB molecules were not released, meaning that the adsorption of RhB was not on the surface of Zr-MOF and RhB@Zr-MOF had been successfully synthesized (Fig. 1a). The PXRD data of RhB@Zr-MOF demonstrate the excellent purity and homogeneity, and are consistent with the simulated data (Fig. 1b). Meanwhile, the IR spectra of Zr-MOF and RhB@Zr-MOF are nearly the same, implying that the framework of Zr-MOF is completely maintained (Fig. S1†). In the TG curves of Zr-MOF and RhB@Zr-MOF, a small difference of 8.6% at 200 °C for the two materials represents the successful loading of RhB (Fig. 1c). The mass fraction of RhB in RhB@Zr-MOF is 5.6%, and this value was obtained by comparing the two TG curves with the theoretical value of 61.9%, which represents the case in which all of the RhB molecules entered the channels of Zr-MOF. The N_2_ adsorption isotherms imply that RhB@Zr-MOF is still a porous material, with a Brunauer–Emmett–Teller (BET) surface area of 117 m^2^ g^−1^. This value is smaller than that of Zr-MOF, which has a BET surface area of 613 m^2^ g^−1^ (Fig. S2†).^43^ All the phenomena listed above indicate that RhB luminescent molecules have been encapsulated into the channels of Zr-MOF to successfully construct the novel porous material RhB@Zr-MOF.

### Photoluminescence properties of Zr-MOF and RhB@Zr-MOF

**Fig. 2:**
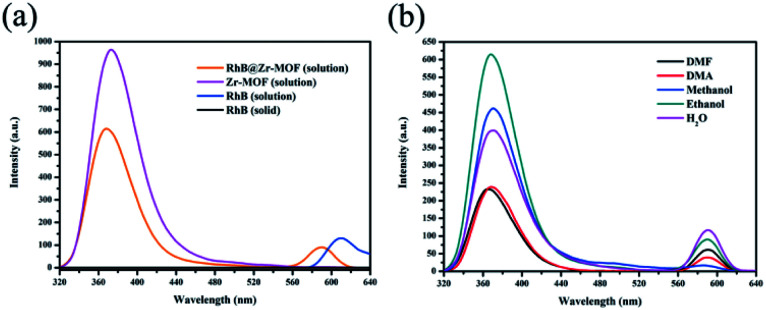
(a) Luminescence emission spectra of Zr-MOF (ethanol solution, 1 g L^−1^), RhB@Zr-MOF (ethanol solution, 1 g L^−1^), RhB (solid state) and RhB (ethanol solution, 1 g L^−1^). (b) Luminescence emission spectra and emission intensities of RhB@Zr-MOF in different solvents.

The luminescence emission spectra of Zr-MOF and RhB@Zr-MOF are shown in Fig. 2a, which were obtained on a fluorescence spectrophotometer using a 1% attenuator. The main emission of Zr-MOF in ethanol solution (1 g L^−1^) is at 372 nm (*λ*_ex_ = 300 nm), displaying a single emission. The main emission of RhB in ethanol solution (1 g L^−1^) is at 610 nm (*λ*_ex_ = 300 nm), also displaying a single emission, while the solid state of RhB exhibits no fluorescence, which may be due to aggregation quenching effects.^51^ After RhB had been encapsulated in the channels of Zr-MOF, the main emissions of RhB@Zr-MOF in ethanol solution (1 g L^−1^) were at 368 nm, originating from Zr-MOF, and 590 nm, originating from RhB molecules (*λ*_ex_ = 300 nm). These two emissions are both slightly blue-shifted compared with those of Zr-MOF and RhB.

In order to detect the capability of RhB@Zr-MOF for small-molecule sensing, the luminescence properties of RhB@Zr-MOF in various solvent solutions were investigated. As shown in Fig. 2b, the solvent effect is the main factor determining the emission intensities of RhB@Zr-MOF. The emission bands are visible at almost the same wavelengths in DMF, DMA, methanol, ethanol and H_2_O. The different degrees of quenching effects may be attributed to the diverse interaction forces between RhB@Zr-MOF and the solvents used, further influencing the electron transfer process.^52^

As a potential sensor, RhB@Zr-MOF with dual-emission properties was applied to detect cations, nitro explosives and pesticides as analytes. Different types and concentrations of analytes can cause different degrees of fluorescence enhancement or quenching. RhB@Zr-MOF possesses a strong emission peak at 368 nm and a weak emission peak at 590 nm, allowing it to serve as a self-calibrating sensor. Here, the relative peak-height, which means the difference of intensities between the strong and the weak peak values, was used to represent the detection signals (Fig. S3†).

### Sensing cations

**Fig. 3:**
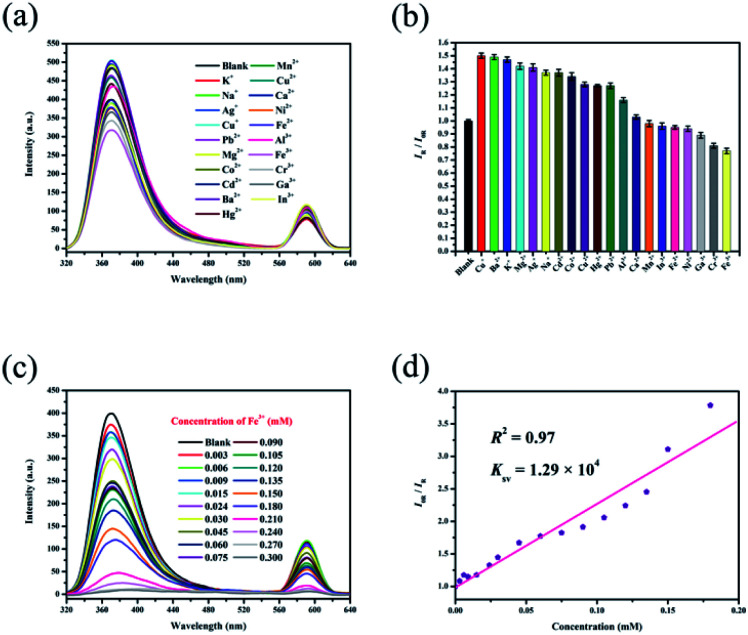
(a) Luminescence emission spectra of RhB@Zr-MOF with different cations in H_2_O. (b) Histogram of relative luminescence intensities of RhB@Zr-MOF immersed in H_2_O containing different cations with a concentration of 0.030 mM (*λ*_ex_ = 300 nm). (c) Luminescence emission spectra of RhB@Zr-MOF at varying Fe^3+^ concentrations (*λ*_ex_ = 300 nm). (d) Stern–Volmer plot of RhB@Zr-MOF for Fe^3+^.

The luminescence emission spectra show that the two main emissions of RhB@Zr-MOF in H_2_O are relatively far apart and do not overlap, making this material suitable for sensing cations and allowing it to avoid mutual interferences. The relative peak-height of RhB@Zr-MOF in solvent with no analytes is defined as *I*_0R_, and with analytes it is defined as *I*_R_. *I*_R_/*I*_0R_, which represents the relative luminescence intensity (RLI). Aqueous solutions of K^+^, Na^+^, Ag^+^, Cu^+^, Pb^2+^, Mg^2+^, Co^2+^, Cd^2+^, Ba^2+^, Hg^2+^, Mn^2+^, Cu^2+^, Ca^2+^, Ni^2+^, Fe^2+^, Al^3+^, Fe^3+^, Cr^3+^, Ga^3+^ and In^3+^ were studied, and different cations show different quenching efficiencies compared with a blank sample (Fig. 3a). The RLI value of the blank sample is 1, while the RLI values of samples of Cu^+^, Ba^2+^, K^+^, Mg^2+^, Ag^+^, Na^+^, Cd^2+^, Co^2+^, Cu^2+^, Hg^2+^, Pb^2+^, Al^3+^ and Ca^2+^ range from 1.03 to 1.50, as shown in Table S1,† displaying fluorescence enhancement to different degrees. The mechanism may be the strong exciplex formation between RhB@Zr-MOF and these inorganic ions.^53^ It is possible that there are strong electrostatic interactions between the organic ligands with rich π electrons acting as electron donors and inorganic ions with high charge-to-size ratios.^54^ The fluorescence enhancement may be explained by the donor–acceptor electron transfer mechanism. Generally, if the excited electrons transfer from a high-lying π*-type LUMO to the conduction band (CB) of RhB@Zr-MOF, fluorescence intensity will arise.^55^ The RLI values of Mn^2+^, In^3+^, Fe^2+^, Ni^2+^, Ga^3+^, Cr^3+^ and Fe^3+^ range from 0.77 to 0.98, displaying fluorescence quenching (Fig. 3b). This result demonstrates that RhB@Zr-MOF is extremely sensitive to trivalent metal ions (Fe^3+^, Cr^3+^ and Ga^3+^), especially Fe^3+^. Next, the titration method was adopted for RhB@Zr-MOF sensing Fe^3+^ at different concentrations ranging from 0.003 mM to 0.300 mM (Fig. 3c). The intensity decreases sharply with increasing concentration. The quenching efficiency for Fe^3+^ is nearly 100% at a concentration of 0.300 mM. In order to quantify the quenching efficiencies, the Stern–Volmer (SV) equation, (*I*_0R_/*I*_R_) = *K*_sv_[A] + 1, was adopted, where [A] is the molar concentration, and *K*_sv_ is the quenching constant (M^−1^).^56^ The *K*_sv_ of RhB@Zr-MOF for sensing Fe^3+^ is 1.29 × 10^4^ M^−1^, and the plot displays a broad concentration range, implying that RhB@Zr-MOF is a good candidate for detecting Fe^3+^ (Fig. 3d). The equation LOD = 3*σ*/*S* was adopted to calculated the LOD for sensing Fe^3+^, in which *S* is the slope of the calibration, and *σ* represents the standard deviation of the response at the lowest concentration.^57^ The LOD of RhB@Zr-MOF for detecting Fe^3+^ is 1.6 μM.

It is known that outstanding recyclability is important for chemical sensors. RhB@Zr-MOF can be regenerated by centrifuging the suspension after detecting Fe^3+^, washing with DMF and H_2_O several times, and drying at ambient temperature. Remarkably, the original intensities remained nearly unchanged, and the same samples of RhB@Zr-MOF were reused for five cycles (Fig. S4†), demonstrating that RhB@Zr-MOF can serve as a practical fluorescence sensor for Fe^3+^ detection. Meanwhile, the UV-vis spectra were studied to investigate the mechanism of sensing Fe^3+^ (Fig. S5†). We have chosen K^+^, Na^+^, Hg^2+^, Mn^2+^, Fe^3+^ and In^3+^ as examples. It is obvious that Fe^3+^ showed light adsorption ranging from 200 to 250 nm. The degrees of adsorption for the six cations in UV-vis spectra exactly match well with their quenching efficiencies from the luminescence emission spectra.

### Sensing nitro explosives

**Fig. 4:**
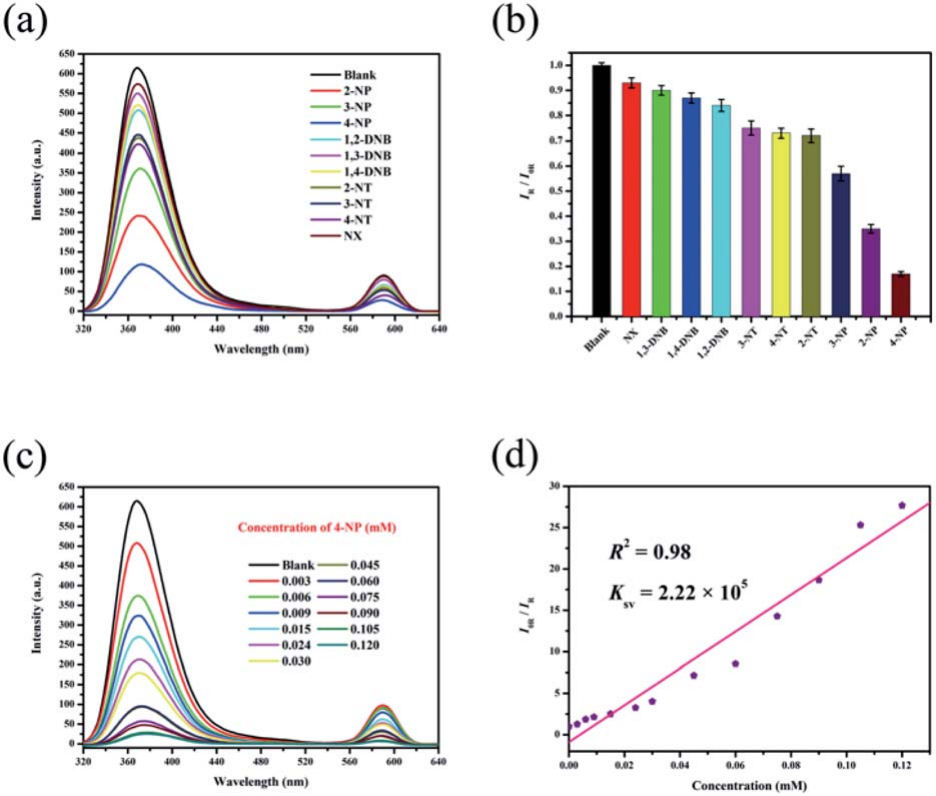
(a) Luminescence emission spectra of RhB@Zr-MOF with different nitro explosives in ethanol solutions. (b) Histogram of relative luminescence intensities of RhB@Zr-MOF immersed in ethanol solutions containing different nitro explosives at a concentration of 0.030 mM (*λ*_ex_ = 300 nm). (c) Luminescence emission spectra of RhB@Zr-MOF at varying 4-NP concentrations (*λ*_ex_ = 300 nm). (d) Stern–Volmer plot of RhB@Zr-MOF for 4-NP.

Ethanol solutions of nitro explosives (1,2-DNB, 1,3-DNB, 1,4-DNB, 2-NP, 3-NP, 4-NP, 2-NT, 3-NT, 4-NT and NX) were then studied, and these different analytes show different quenching efficiencies compared with a blank sample (Fig. 4a). The RLI values of all the nitro explosives range from 0.17 to 0.93 (Table S2†), displaying fluorescence quenching (Fig. 4b). This result demonstrates that RhB@Zr-MOF is extremely sensitive to 4-NP. Next, the titration method was adopted for RhB@Zr-MOF sensing 4-NP at different concentrations ranging from 0.003 mM to 0.120 mM (Fig. 4c). The intensity decreases sharply with increasing concentration. The quenching efficiency for 4-NP is nearly 100% at a concentration of 0.120 mM. The *K*_sv_ of RhB@Zr-MOF for sensing 4-NP is 2.22 × 10^5^ M^−1^, and the plot displays a broad concentration range, implying that RhB@Zr-MOF can detect 4-NP more rapidly and sensitively than Fe^3+^ (Fig. 4d). The LOD of RhB@Zr-MOF for detecting 4-NP is 0.9 μM.

The same samples of RhB@Zr-MOF were reused for 5 cycles for the detection of 4-NP and the original intensities remained nearly unchanged (Fig. S6†). Meanwhile, the UV-vis spectra were studied to further assess the sensing ability of RhB@Zr-MOF for 4-NP (Fig. S7†).

### Sensing pesticides

**Fig. 5:**
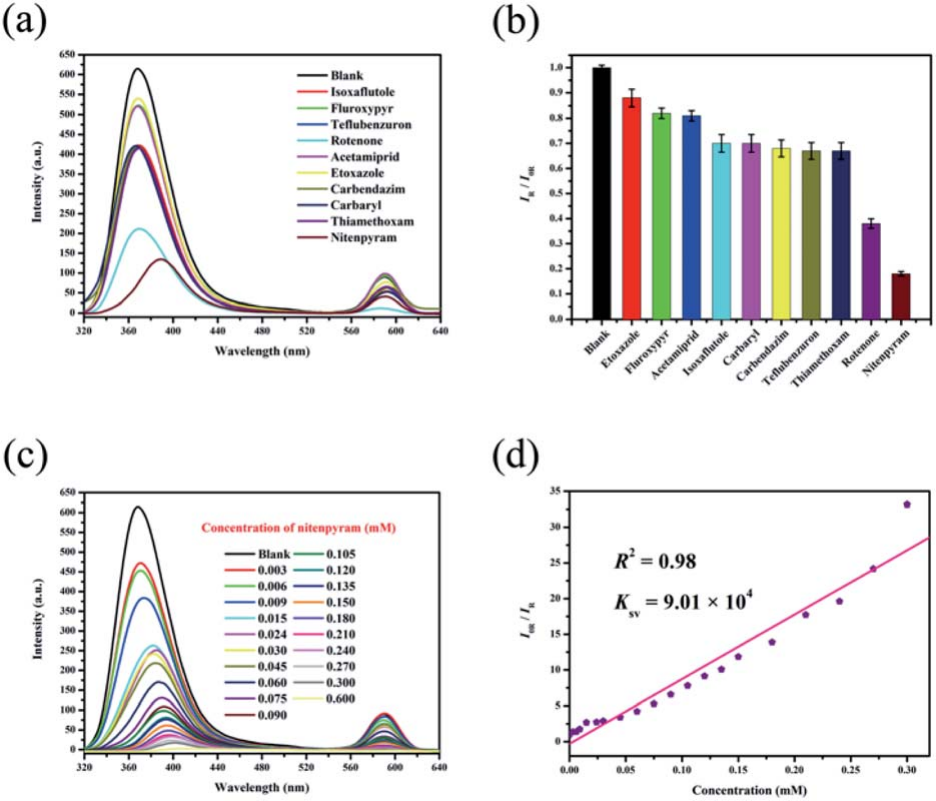
(a) Luminescence emission spectra of RhB@Zr-MOF with different pesticides in ethanol solutions. (b) Histogram of relative luminescence intensities of RhB@Zr-MOF immersed in ethanol solutions containing different pesticides with a concentration of 0.030 mM (*λ*_ex_ = 300 nm). (c) Luminescence emission spectra of RhB@Zr-MOF at varying nitenpyram concentrations (*λ*_ex_ = 300 nm). (d) Stern–Volmer plot of RhB@Zr-MOF for nitenpyram.

Ethanol solutions of isoxaflutole, fluroxypyr, teflubenzuron, rotenone, acetamiprid, etoxazole, carbendazim, carbaryl, thiamethoxam and nitenpyram (Fig. S8†) were then studied. The different pesticides show different quenching efficiencies compared with a blank sample (Fig. 5a). The RLI values of all of the pesticides range from 0.18 to 0.88 (Table S3†), displaying fluorescence quenching (Fig. 5b), and RhB@Zr-MOF is extremely sensitive to nitenpyram. Next, the titration method was adopted for RhB@Zr-MOF sensing nitenpyram with different concentrations ranging from 0.003 mM to 0.600 mM (Fig. 5c). The intensity decreases sharply with increasing concentration. The quenching efficiency for nitenpyram is nearly 100% at a concentration of 0.600 mM. The *K*_sv_ of RhB@Zr-MOF is 9.01 × 10^4^ M^−1^ for detecting nitenpyram, and the plot displays a broad concentration range, implying that RhB@Zr-MOF can detect nitenpyram more rapidly and sensitively than Fe^3+^, but slightly more slowly than 4-NP (Fig. 5d). The LOD of RhB@Zr-MOF for detecting nitenpyram is 0.2 μM.

PXRD data were gathered for RhB@Zr-MOF after removing it from ethanol solutions of nitenpyram *via* centrifugation. The original data of RhB@Zr-MOF match well with these data (Fig. S9†). The same samples of RhB@Zr-MOF were reused for 5 cycles for the detection of nitenpyram and the original intensities remained nearly unchanged (Fig. S10†). Meanwhile, UV-vis spectra were studied to further identify the sensing ability of RhB@Zr-MOF for nitenpyram (Fig. S11†).

### Detecting nitenpyram in real samples

RhB@Zr-MOF was utilized for the detection of nitenpyram in real samples. Water from the Songhua river was selected, and the standard addition method was used to introduce nitenpyram. Nitenpyram with concentrations of 0.3 μM, 3.0 μM, 30.0 μM and 300.0 μM was added to the samples of local water. The data, which are listed in Table 1, imply excellent recovery rates ranging from 96.3% to 101.8% for RhB@Zr-MOF. The RSD values calculated range up to 4.25%. These results demonstrate moderate accuracy and precision.

**Table 1:** Data for RhB@Zr-MOF detecting nitenpyram in real samples

RhB@Zr-MOF	Added (μM)	Found^a^ (μM)	Recovery (%)	RSD^b^ (%)
Water source	0.3	0.289 ± 0.005	96.3 ± 1.7	1.38
	3.0	2.938 ± 0.082	97.9 ± 2.7	2.19
	30.0	30.541 ± 0.621	101.8 ± 2.1	4.25
	300.0	291.6293 ± 3.722	92.2 ± 1.2	3.01

a Average of three repeated measurements of nitenpyram.

b RSD means relative standard deviation.

### Mechanism of detecting cations, nitro explosives and pesticides

**Scheme 1:**
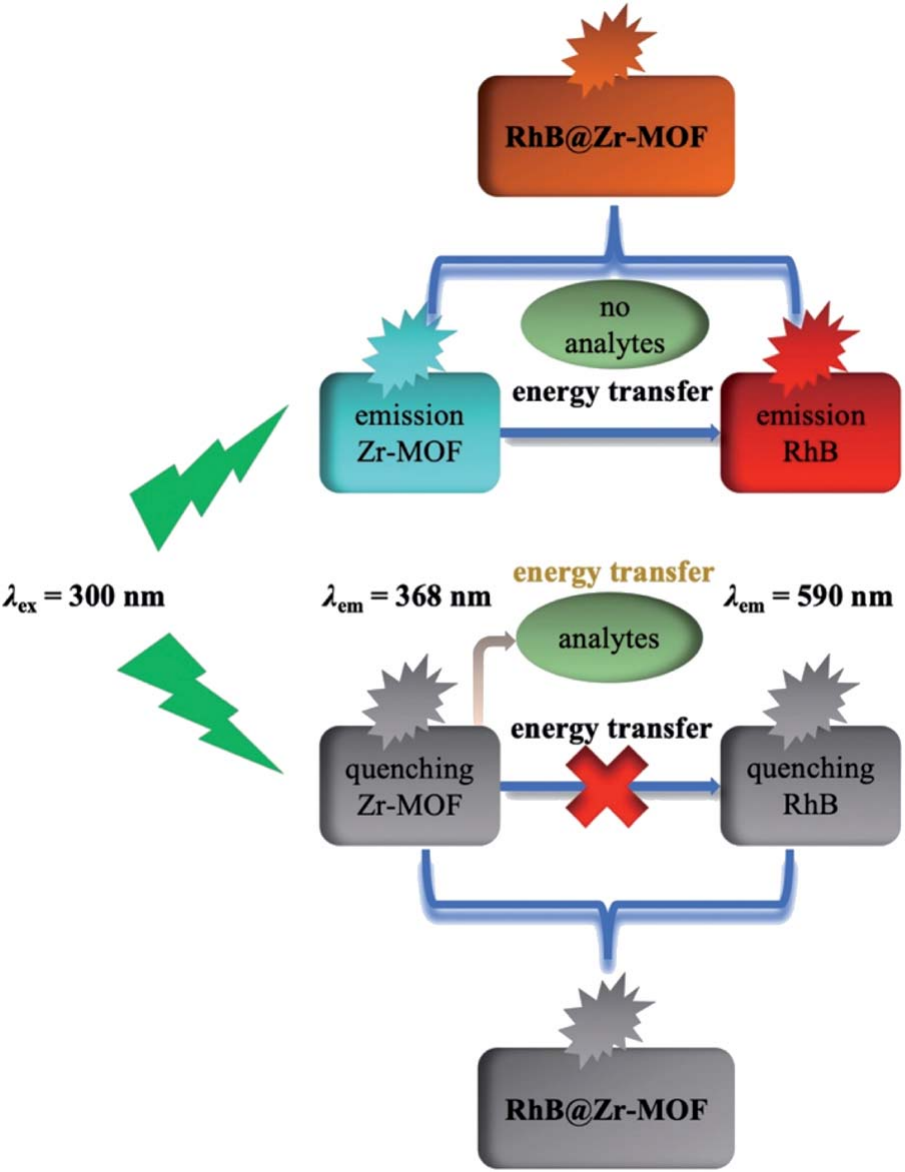
The mechanism of RhB@Zr-MOF as a fluorescence sensor for detecting analytes.

**Fig. 6:**
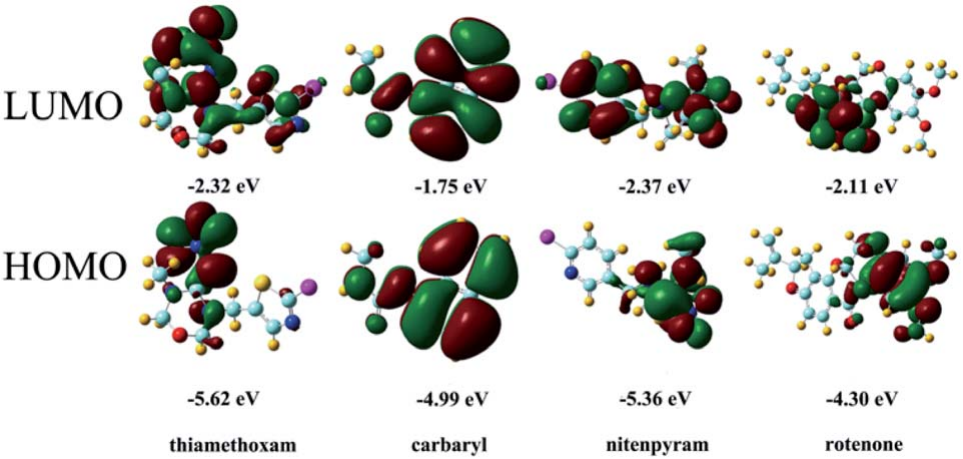
HOMOs and LUMOs of the pesticides listed, with relative energy levels.

The mechanism of analyte detection was investigated thoroughly. From the perspective of resonance energy transfer, the spectral overlap between the emission of this Zr-MOF and the absorption of RhB can be observed, as shown in Fig. S12,† implying the transfer of resonance energy from Zr-MOF to RhB.^58^ To further confirm this phenomenon, the fluorescence lifetimes of RhB and RhB@Zr-MOF were measured. The lifetime is 1.46 ns for RhB in aqueous solution and 5.21 ns for RhB@Zr-MOF in the solid state. The longer lifetime of RhB@Zr-MOF further demonstrates the existence of resonance energy transfer from Zr-MOF to RhB. In the UV-vis spectra, the absorption wavelengths of Fe^3+^, 4-NP and nitenpyram are closer to the emission spectra of Zr-MOF than those of the other analytes ranging from 200 to 800 nm (Fig. S5, S7 and S11†). Obviously, the absorption band of Fe^3+^, 4-NP and nitenpyram can cover the emission of RhB@Zr-MOF resulting from Zr-MOF. The superior spectral overlaps between Zr-MOF and analytes strongly enhance the transfer of resonance energy from Zr-MOF to analytes, which may hinder the process of transfer from Zr-MOF to guest RhB molecules. The two processes lead to the quenching effects of RhB@Zr-MOF at nearly 368 nm and 590 nm (Scheme 1). From the viewpoint of the analytes, the electron-withdrawing groups contribute to the degree of quenching, where the photo-induced electron-transfer direction is from the excited state of RhB@Zr-MOF to the electron-deficient analytes.^59^ Pesticides including carbaryl, thiamethoxam, rotenone and nitenpyram were selected as examples. The HOMO and LUMO orbital energies have also been calculated by DFT (density functional theory), at the B3LYP/6-31G* level, to further study the mechanism (Fig. 6). Nitenpyram shows the lowest LUMO energy level among the four pesticides, demonstrating that nitenpyram with –NO_2_ groups has a relatively higher electron affinity than the other pesticides, resulting in the electrons transferring from the HOMO of RhB@Zr-MOF to the LUMO of the excited state of the pesticide.^60^

## Conclusions

In summary, RhB@Zr-MOF, a self-calibrating fluorescence sensor, has been successfully constructed and has been demonstrated as a multifunctional, efficient sensor for cations, nitro explosives and pesticides. RhB@Zr-MOF reveals superior recyclability and LODs as a dual-emission chemical sensor. The relative peak-height, defined as the difference of intensities between the strong and the weak peak values, has been employed to represent the detection signals. The LOD of RhB@Zr-MOF for detecting Fe^3+^ is 1.6 μM, that for 4-NP is 0.9 μM and that for nitenpyram is 0.2 μM. Furthermore, RhB@Zr-MOF sensors were utilized for detecting pesticides in real samples. Finally, the mechanism for the detection was analyzed to extend the range of use of RhB@Zr-MOF as a multifunctional sensor.

## Conflicts of interest

There are no conflicts to declare.

## Supplementary Material

RA-010-D0RA02843F-s001
