# Supplementary material for: A built-in self-calibrating luminescence sensor based on RhB@Zr-MOF for detection of cations, nitro explosives and pesticides
Source: RSC Adv. 2020 May 20;10(33):19149–56. doi: 10.1039/d0ra02843f (PMC9054042; doi:10.1039/d0ra02843f)
Supplement: RA-010-D0RA02843F-s001 [file RA-010-D0RA02843F-s001.pdf]

Electronic Supplementary Material (ESI) for RSC Advances.
This journal is © The Royal Society of Chemistry 2020

Electronic Supplementary Information (ESI)

A built-in self-calibrating luminescent sensor based on RhB@Zr-MOF for cations, nitro explosives and pesticides detection

Liu Yang[a,§], Yu-Long Liu[a,§], Cheng-Guo Liu[b], Ying Fu[a,] and Fei Ye[a,*]*

[a]Department of Applied Chemistry, College of Science, Northeast Agricultural University, Harbin, 150030, People's Republic of China.

[b]Department of State Assets Management, Northeast Agricultural University, Harbin, 150030, People's Republic of China.

E-mail: fuying@neau.edu.cn; yefei@neau.edu.cn

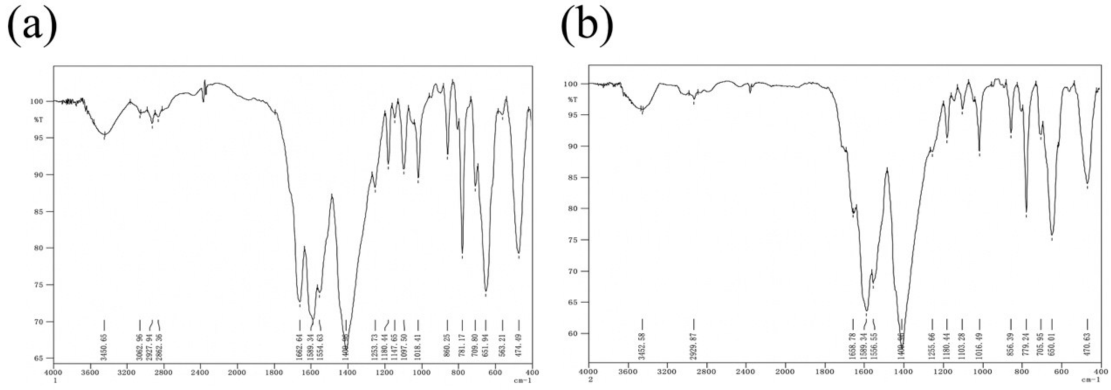

Fig. S1. (a) IR spectra of Zr-MOF. (b) IR spectra of **RhB@Zr-MOF**.

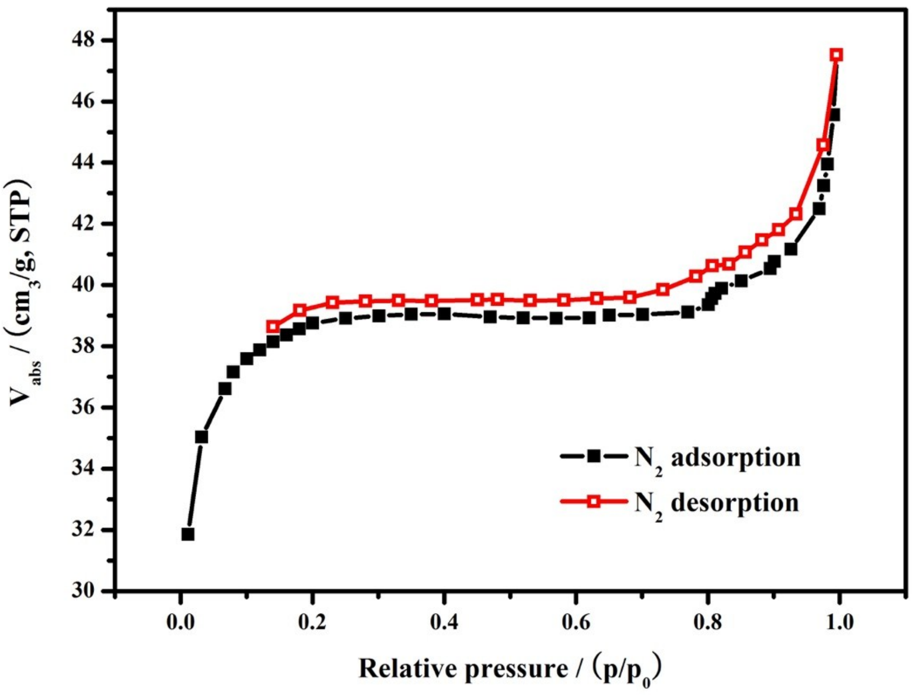

Fig. S2. N_2 adsorption isotherm of **RhB@Zr-MOF** at 77 K.

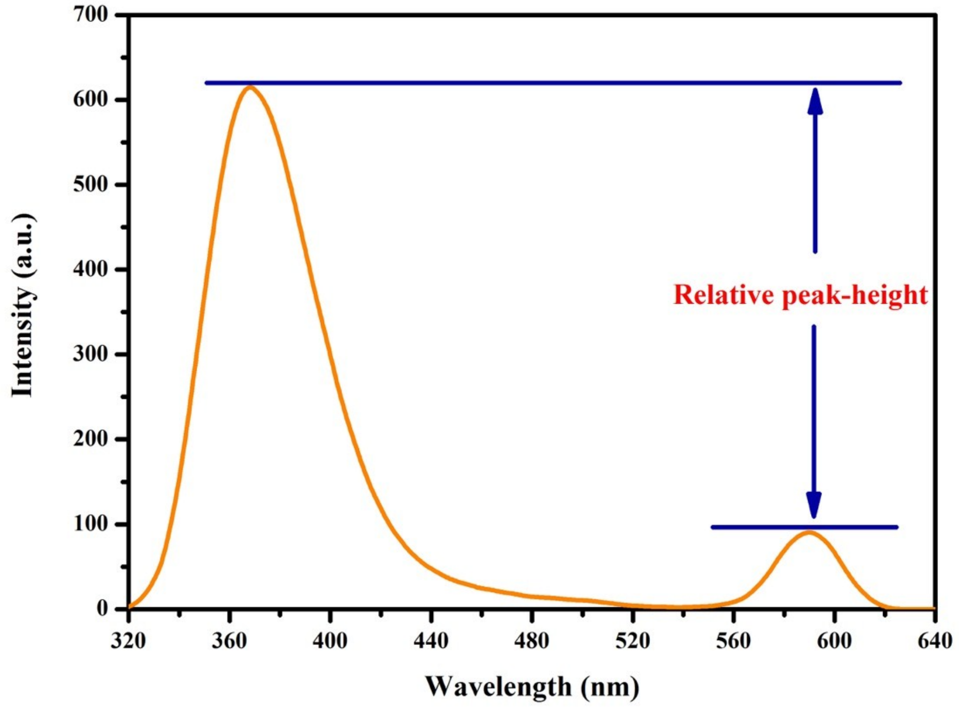

Fig. S3. The definition of relative peak-height.

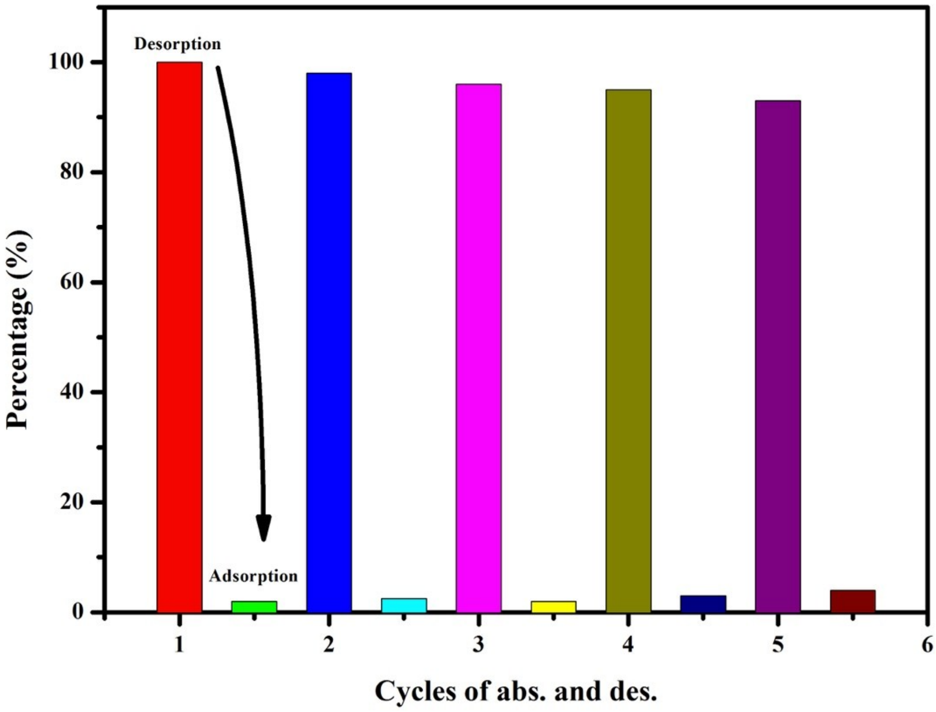

Fig. S4. Recycling test on sensing Fe^{3+} in H_2O by **RhB@Zr-MOF** composite.

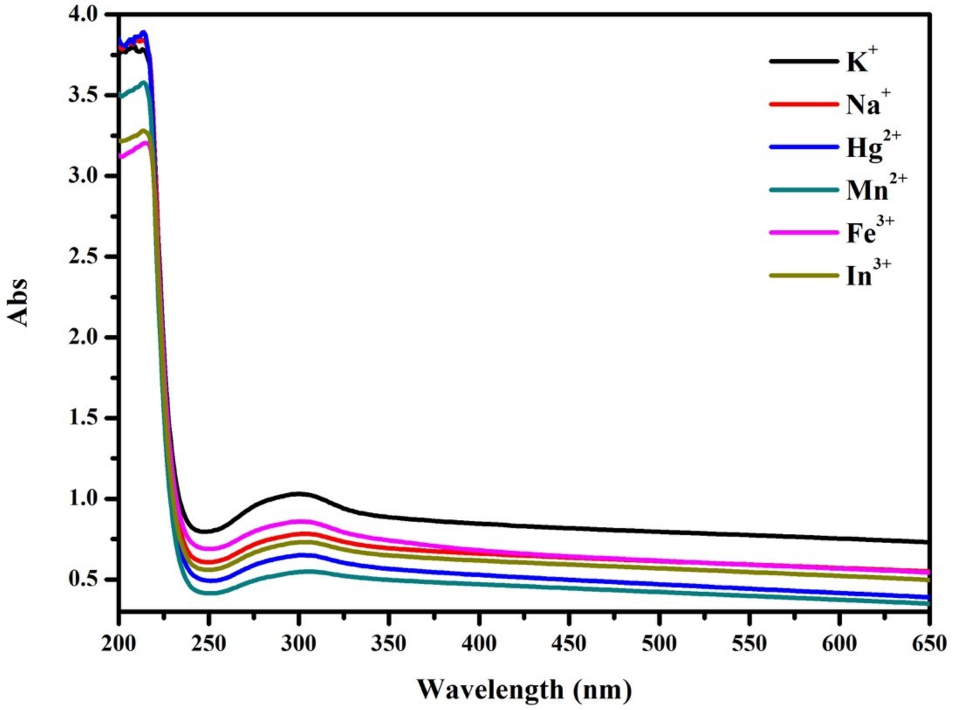

Fig. S5. UV-Vis spectra of **RhB@Zr-MOF** with different cations in H_2O.

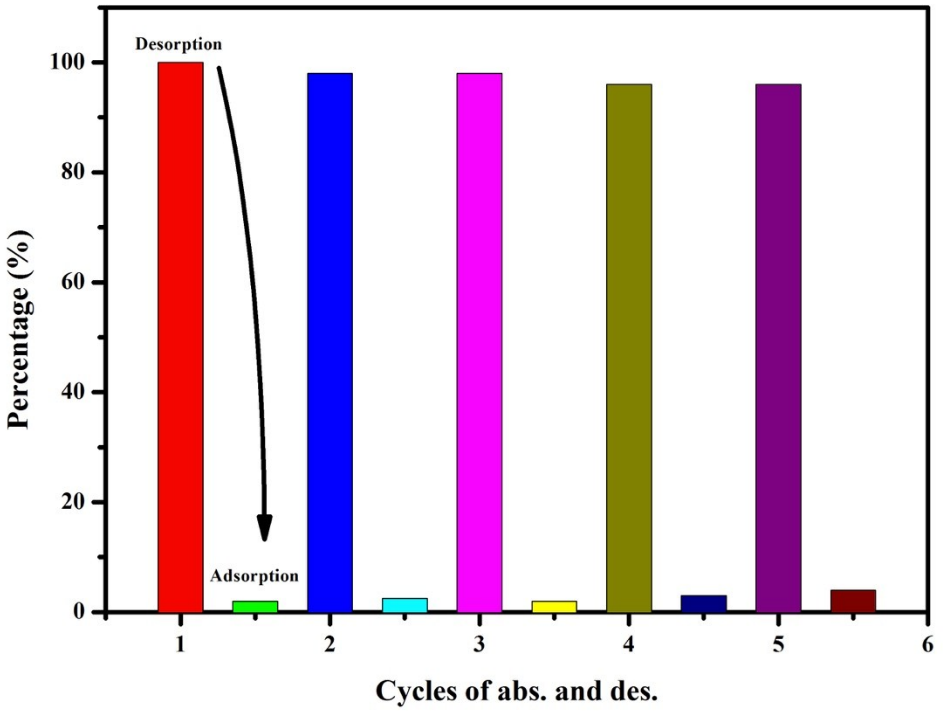

Fig. S6. Recycling test on sensing 4-NP in ethanol solution by **RhB@Zr-MOF** composite.

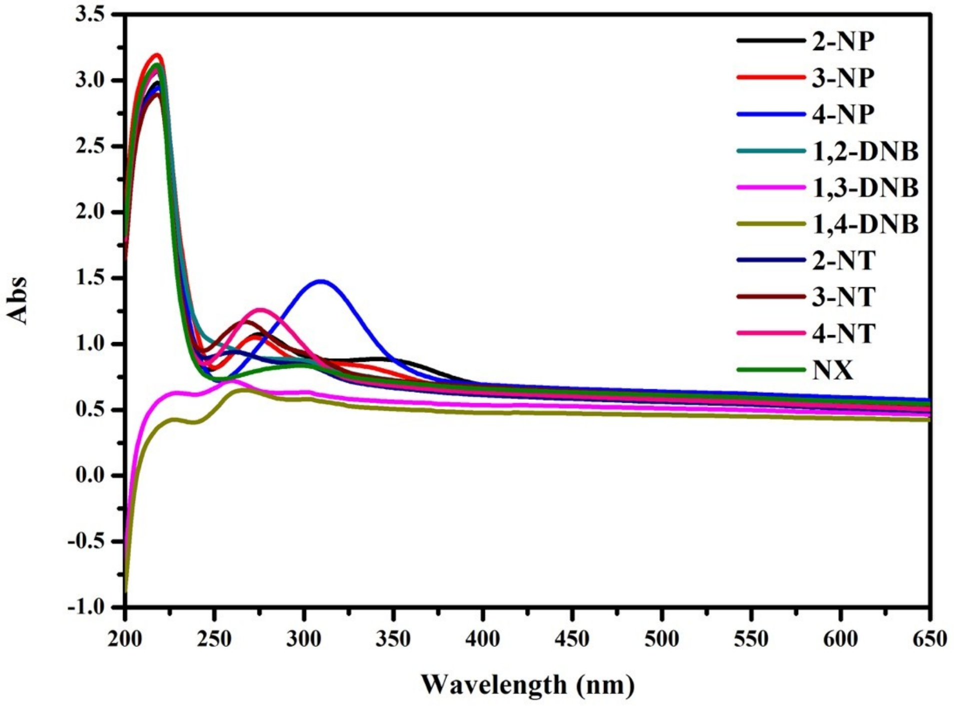

Fig. S7. UV-Vis spectra of **RhB@Zr-MOF** with different nitro explosive in ethanol solution.

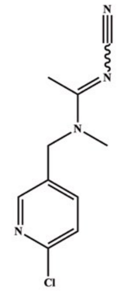

acetamiprid

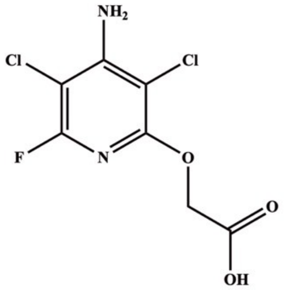

fluroxypyr

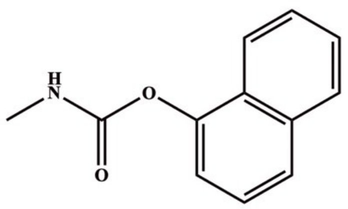

carbaryl

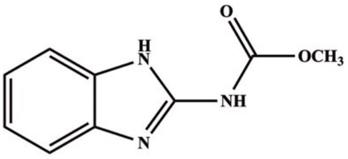

carbendazim

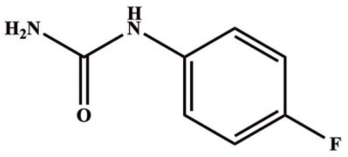

teflubenzuron

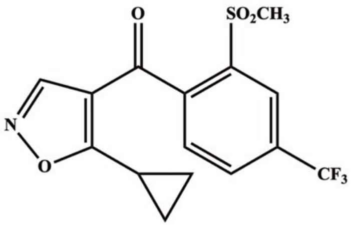

isoxaflutole

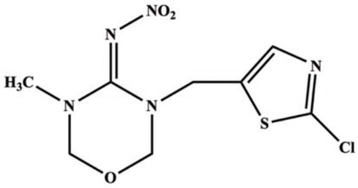

thiamethoxam

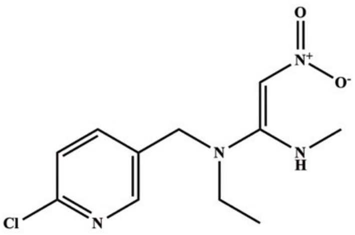

nitenpyram

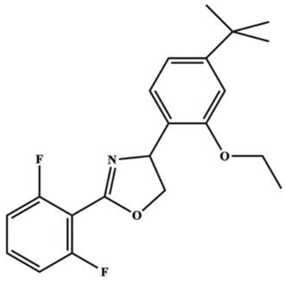

etoxazole

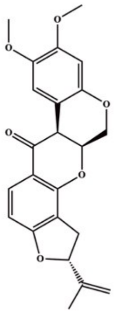

rotenone

Fig. S8. The structures of the pesticides used in this work.

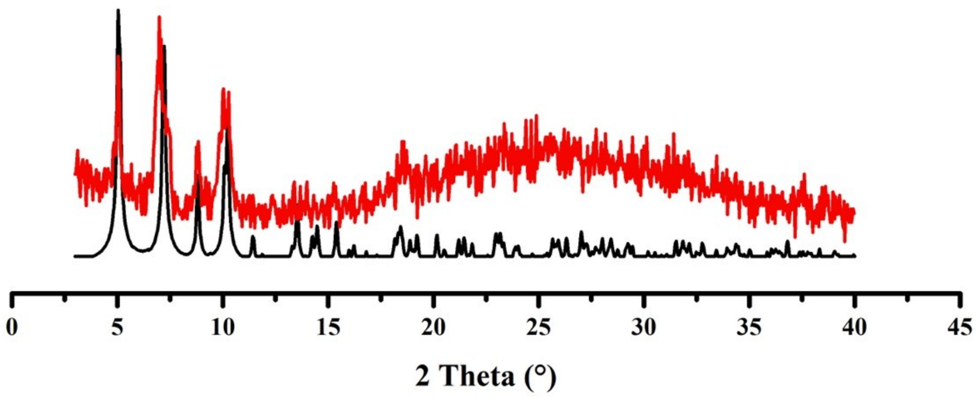

Fig. S9. PXRD powder diffraction patterns of simulated (black), and **RhB@Zr-MOF** centrifugated from ethanol solutions of pesticides (red).

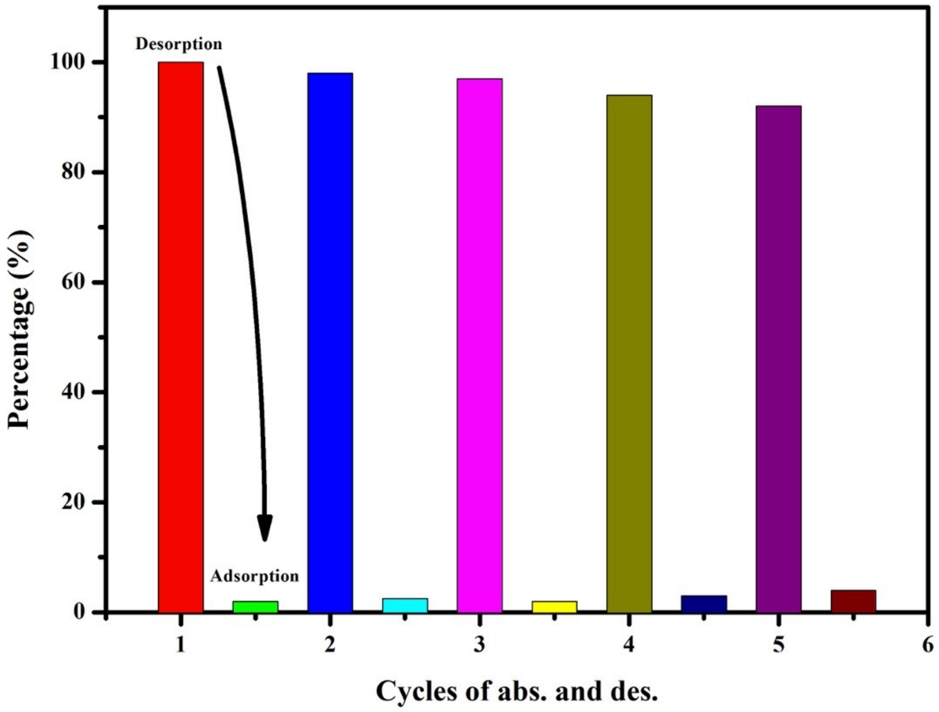

Fig. S10. Recycling test on sensing nitenpyram in ethanol solution by **RhB@Zr-MOF** composite.

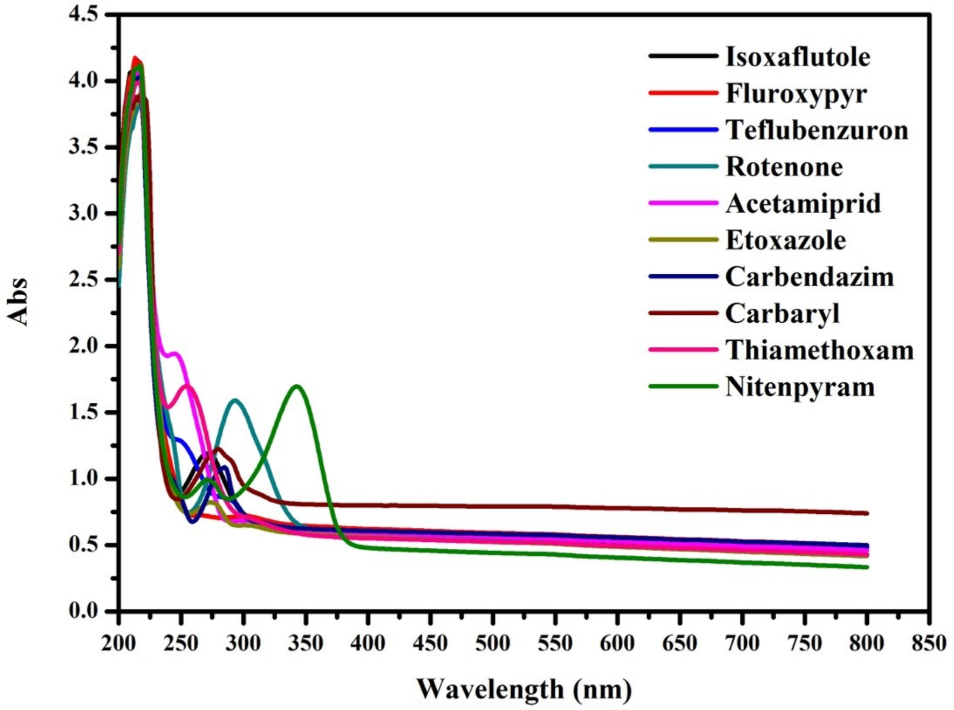

Fig. S11. UV-Vis spectra of **RhB@Zr-MOF** with different pesticides in ethanol solution.

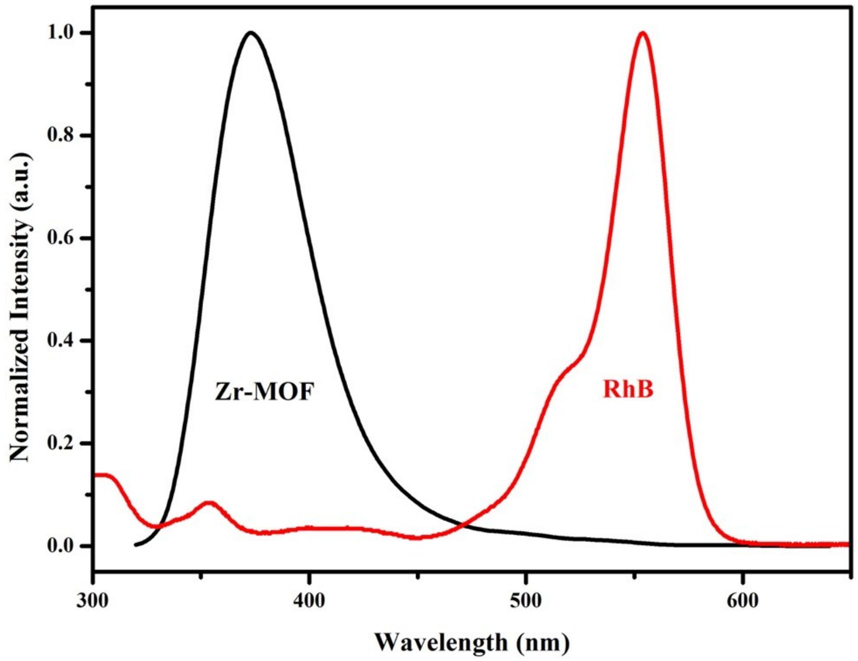

Fig. S12. Normalized emission spectrum of Zr-MOF in the solid state (black) and absorption spectrum of RhB in aqueous solution (red, 0.01 g L^{-1}).

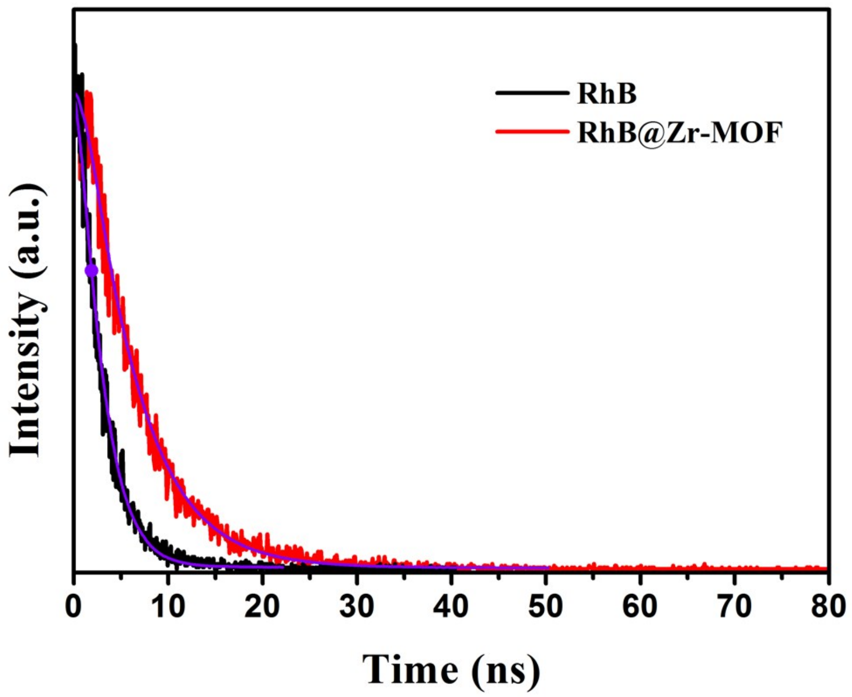

Fig. S13. Luminescence decay curves of RhB in aqueous solution (10 mg L^{-1}) and **RhB@Zr-MOF** in the solid state.

Analytes	Cu^{+}	Ba^{2+}	K^{+}	Mg^{2+}	Ag^{+}	Na^{+}	Cd^{2+}	Co^{2+}	Cu^{2+}	Hg^{2+}
RLI value	1.50	1.49	1.47	1.42	1.41	1.37	1.37	1.34	1.28	1.27
Analytes	Pb^{2+}	Al^{3+}	Ca^{2+}	Mn^{2+}	In^{3+}	Fe^{2+}	Ni^{2+}	Ga^{3+}	Cr^{3+}	Fe^{3+}
RLI value	1.27	1.16	1.03	0.98	0.96	0.95	0.94	0.89	0.81	0.77

Table S1. The RLI values of different cations.

Analytes	NX	1,3-DNB	1,4-DNB	1,2-DNB	3-NT
RLI value	0.93	0.90	0.87	0.84	0.75
Analytes	4-NT	2-NT	3-NP	2-NP	4-NP
RLI value	0.73	0.72	0.57	0.35	0.17

Table S2. The RLI values of different nitro explosives.

Analytes	Etoxazole	Fluroxypyr	Acetamiprid	Isoxaflutole	Carbaryl
RLI value	0.88	0.82	0.81	0.70	0.70
Analytes	Carbendazim	Teflubenzuron	Thiamethoxam	Rotenone	Nitenpyram
RLI value	0.68	0.67	0.67	0.38	0.18

Table S3. The RLI values of different pesticides.
